# Differential microbiota network in gingival tissues between periodontitis and periodontitis with diabetes

**DOI:** 10.3389/fcimb.2022.1061125

**Published:** 2022-12-02

**Authors:** Yeuni Yu, Hyun-Joo Kim, Jae-Min Song, Junho Kang, Hansong Lee, Hae Ryoun Park, Yun Hak Kim

**Affiliations:** ^1^ Biomedical Research Institute, School of Medicine, Pusan National University, Yangsan, South Korea; ^2^ Department of Periodontology, Dental and Life Science Institute, School of Dentistry, Pusan National University, Yangsan, South Korea; ^3^ Department of Periodontology and Dental Research Institute, Pusan National University Dental Hospital, Yangsan, South Korea; ^4^ Periodontal Disease Signaling Network Research Center, School of Dentistry, Pusan National University, Yangsan, South Korea; ^5^ Department of Oral and Maxillofacial Surgery, School of Dentistry, Pusan National University, Yangsan, South Korea; ^6^ Convergence Medical Sciences, Pusan National University, Yangsan, South Korea; ^7^ Department of Oral Pathology, School of Dentistry, Pusan National University, Yangsan, South Korea; ^8^ Department of Anatomy, School of Medicine, Pusan National University, Yangsan, South Korea; ^9^ Department of Biomedical Informatics, School of Medicine, Pusan National University, Yangsan, South Korea

**Keywords:** periodontitis, diabetes mellitus, microbiota, whole genome sequencing, gingiva

## Abstract

Periodontitis and diabetes mellitus (DM) have a bidirectional relationship. Periodontitis is initiated by dysbiosis of oral microorganisms, and in particular, the characteristics of the microorganisms that have penetrated the tissue are directly related to the disease; therefore, we investigated the effect of DM on intragingival microbial profiling of patients with periodontitis. A total of 39 subjects were recruited and divided into three groups in this case control study as follows: healthy (NA, 10), periodontitis only (PD, 18), and periodontitis with DM (PD_DM, 11). Gingival tissue was collected, DNA was extracted, and whole-genome sequencing was performed. PD and PD_DM showed different characteristics from NA in diversity and composition of the microbial community; however, no difference was found between the PD nad PD_DM. PD_DM showed discriminatory characteristics for PD in the network analysis. PD showed a network structure in which six species were connected, including three red complex species, and PD_DM’s network was more closely connected and expanded, with six additional species added to the PD network. Although DM did not significantly affect α- and β-diversity or abundance of phyla and genera of microbiota that invaded the gingival tissue of patients with periodontitis, DM will affect the progression of periodontitis by strengthening the bacterial network in the gingival tissue.

## Background

Periodontitis and diabetes mellitus (DM) are both chronic non-communicable inflammatory diseases that are affected by a multifactorial etiology in the development and progression of diseases, and their bidirectional relationship has been established for a long time (Mealey and Oates, 2006). In 2017, according to the consensus report of the International Diabetic Federation and the European Federation of Periodontology, periodontitis and DM were risk factors for each other (Sanz et al., 2018). Since both periodontitis and DM have a high global prevalence, and it requires immense social and economic efforts to control these disease (Eke et al., 2016; Harreiter and Roden, 2019), it is necessary to elucidate the relationship between the two diseases for targeted screening and early detection of both the diseases in risk groups. According to the extant literature, periodontitis and DM are mechanistically linked through an increase in pro-inflammatory mediators such as interleukin (IL)-1β, tumor necrosis factor, IL-6, and oxidative stress (Sanz et al., 2018).

Periodontitis and DM are reported to be associated with changes in oral and gut microbiota, respectively, when compared to healthy conditions (Teles et al., 2013; Lambeth et al., 2015). Salivary microbiome analysis according to the presence or absence of diabetes in patients with periodontitis revealed that a change in genera abundance was observed under diabetic condition (Sabharwal et al., 2019). DM can alter host responses, which can also cause changes in the oral environment. For example, increased blood sugar levels can lead to increased glucose levels in the gingival crevicular fluid and saliva (Ficara et al., 1975; Satish et al., 2014). Changes in nutrition available to microorganisms cause environmental alterations in the microbial community, which results in a more acidic and anaerobic state, which ultimately leads to microbial dysbiosis (Takahashi et al., 1997).

In periodontitis, a dysbiotic microbial change leads to an increase in the pathogenicity of the microbial community, and some species, including *Prevotella intermedia*, *Treponema denticola*, and *Porphyromonas gingivalis* are known to infiltrate tissues during the disease process (Ji et al., 2015). The increase in bacterial infiltration into the gingival tissue is correlated with the increase in T cells in the tissue and loss of alveolar bone. Therefore, to clearly identify the bidirectional relationship between periodontitis and DM, it is necessary to determine whether there is a difference in the microorganisms infiltrating the periodontal tissue in patients with periodontitis depending on the presence or absence of DM. In the past, electron microscopy, immunofluorescence, immunohistochemistry, and *in situ* hybridization methods have been used to detect bacteria in tissues (Peković and Fillery, 1984; Saglie et al., 1988; Kim et al., 2010); however, these methods are limited in their ability to set candidate biomarkers (e.g. cytokines, metabolites, and etc.) detect bacteria. Therefore, in this study, we investigated the effect of DM on the microbiome invading the gingival tissue of patients with periodontitis using a sequencing method that an unbiased manner.

## Methods

### Study populations and clinical examination

A total of 39 subjects were recruited from the Department of Periodontics, Pusan National University Dental Hospital (Yangsan, Korea). The general inclusion criteria for subjects were as follows: 1) they were free of systemic diseases that could influence the prognosis of periodontitis except DM; 2) were not pregnant or breastfeeding; 3) had not taken systemic antibiotics, anti-inflammatory drugs (steroid or non-steroidal anti-inflammatory drugs), or oral antiseptic agents within 6 months; 4) had not received periodontal therapy in the last 3 months; 5) did not have any acute infection or chronic mucosal lesions of the oral cavity; 6) had at least 20 teeth; 7) were non-smokers; and 8) had signed an informed consent form.

Complete medical and dental histories were collected on the first day of the visit. The presence or absence of DM was determined based on the patient medical history. Subjects with DM (type 2) were diagnosed with HbA1c ≥ 6.5%, fasting plasma glucose test ≥ 7.0 mM/L, or oral glucose tolerance test ≥ 11.1 mM/L (Anonymous, 2020). Periodontal diagnosis followed the guidelines presented by the 2017 World Workshop on the Classification of Periodontal and Peri-implant Diseases and Conditions (Caton et al., 2018). This study included periodontally healthy subjects and those with stage II or III periodontitis. Periodontal health was defined as < 10% bleeding sites with probing depths of ≤ 3 mm. Staging of periodontitis relied on the severity, which was classified according to the following criteria: 1) stage IIa) interdental clinical attachment loss of 3–4 mm, b) radiographic bone loss extending between 15% and 33% of the root length, c) maximum probing depth ≤ 5 mm, and d) no tooth loss due to periodontitis and 2) stage IIIa) interdental clinical attachment loss ≥ 5 mm, b) radiographic bone loss extending to the mid-third of the root and beyond, and c) maximum probing depth ≥ 6 mm, and d) tooth loss due to periodontitis of ≤ 4 teeth. Patients with grade C disease, which indicates a rapid rate of progression or early onset disease, were excluded from the study. Periodontal status was evaluated by a periodontal specialist, and the following parameters were recorded: plaque index, probing depth, clinical attachment level, and bleeding on probing (six sites per tooth).

The subjects were divided into three groups: NA (10 subjects without periodontitis and DM), PD (18 subjects with periodontitis who were in good general health), and PD_DM (11 subjects with periodontitis who had type 2 DM). All subjects provided written consent to participate in this study, and the study protocol was approved by the Institutional Review Board of Pusan National University Dental Hospital (PNUDH-2017-023).

### Collection of gingival tissue samples

A gingival tissue sample, including the pocket epithelium and underlying connective tissue, was taken from each patient. Tissues were collected during periodontal flap surgery in subjects with periodontitis (PD and PD_DM groups). In subjects without periodontitis (NA group), the tissue was collected during the crown lengthening procedure or extraction due to non-periodontal problems. The gingival tissue obtained was washed with sterile normal saline solution to remove any blood clots or detached plaque on the tissue surface.

### Whole genome sequencing (WGS)

The genomic DNA was fragmented using Frag enzyme (MGI, Shenzhen, China) to DNA fragments between 100 and ∼1,000 bp, which was suitable for PE150 sequencing according to the manufacturer’s instructions (MGI FS DNA library prep set, cat No. 1,000,005,256). The fragmented DNA was selected to be between 300 and 500 bp using clean DNA beads (MGI, Shenzhen, China). The selected DNA fragments were repaired to obtain a blunt end and modified at the 3′ end to obtain dATP as a sticky end. The dTTP-tailed adapter sequence was ligated at both ends of the DNA fragments. The ligation product was then amplified for seven cycles and subjected to the following single-strand circularization process: The polymerase chain reaction (PCR) product was heat-denatured with a special molecule that was reverse-complemented to one special strand of the PCR product, and the single-strand molecule was ligated using DNA ligase. The remaining linear molecule was digested with an exonuclease to obtain a single-strand circular DNA library. We sequenced the DNA library using DNBSEQ-T7 (DNBSEQ-T7, RRID : SCR_017981) with a PE read length of 150 bp. We used FastQC v0.11.8 (FastQC, RRID : SCR_014583) to assess the overall sequencing quality of the MGI sequencing platforms.

### Identification and quantification of the microbiome: Kraken2

Sequencing reads that did not align with known human reference genomes (based on mapping information in raw BAM files) were mapped against all known bacterial, archaeal, and viral microbial genomes from the National Center for Biotechnology Information (NCBI) database (Federhen, 2012) using the ultrafast Kraken2 algorithm (Wood and Salzberg, 2014). Kraken2 reports were combined into taxonomic read-abundance tables with Bracken. Newly estimated taxa count through the Bayesian model were used in the analysis and taxa less than 10 counts, the default of the bracken analysis, were removed. Abundance tables of bacteria were used to calculate diversity at the species level in R (version 4.0.3) using the vegan library. To identify the bacteria that distinguish the group, we used Aldex2 (Fernandes et al., 2013). The p-value of each test was adjusted in the false discovery rate (FDR) using the Benjamini–Hochberg algorithm, and a threshold of p-value lower than 0.05 was applied to determine the significance.

### Network analysis

For the networking approach, we inferred co-occurrence networks using the SparCC algorithm to calculate the correlation strength and significance of all variables. A co-occurrence event was considered in the network if the correlation was > |0.6|.

## Results

This study analyzed the microbial community by mapping reads that were not mapped to the human reference to the NCBI database using Kraken2 and Braken. The average proportion of microbes in the samples was 0.16% (**Supplementary Table 1**).

### Comparison of NA, PD, and PD_DM microbe communities

**Figure 1:**
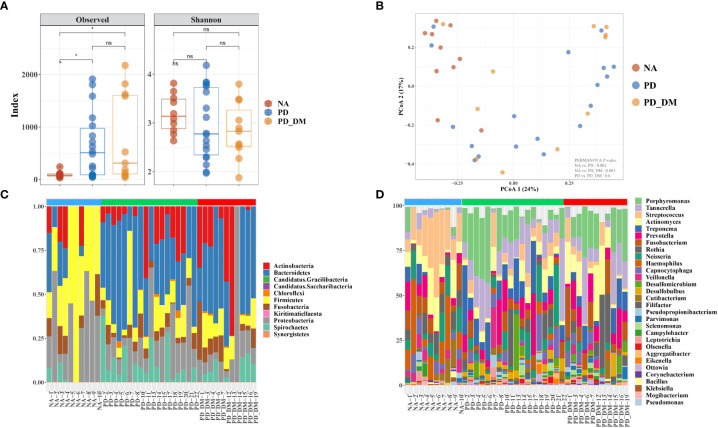
Compositional distinction of oral microbiota between NA, PD, and PD_DM. **(A)** The comparison of oral microbiota alpha diversity between each group, including species richness (represented by Chao1) and evenness (represented by Shannon and pielou). **(B)** The principal component analysis (PCA) plot is shown generated by applying phyloseq to the species level count data. Each point represents one sample, and the samples are colored by status. The percentage of variation explained by each principal component is depicted on each axis. Stacked bar-plot representation of microbiota compositions during NA, PD, and PD_DM, with taxonomic features collapsed at the level of **(C)** phyla and **(D)** genera. The genera represent the top 30 strains. Blue bar, NA; green bar, PD; red bar, PD_DM. *p < 0.05; ns, no significance.

The alpha-diversity indices showed different species richness in terms of the Chao1 index in NA, PD, and PD_DM, while this index was significantly higher in PD and PD_DM. Conversely, species evenness in terms of Shannon was slightly higher in NA (**Figure 1A**). The analysis of beta diversity demonstrated distinct signatures of the oral microbiome in all three groups (**Figure 1B**, **Supplementary Figure 1**). As evident from the principal coordinate analysis (PCoA) of the Bray–Curtis dissimilarity index, the PD and PD_DM samples clustered closely together (PERMANOVA, p = 0.6), while the NA samples constituted slightly separated clusters (PERMANOVA, p < 0.001). At the phylum level, the microbiome composition appeared to be relatively similar in PD and PD_DM, whereas NA demonstrated a distinct composition compared with the other two groups (**Figure 1C**). PD and PD_DM had a significantly higher (p < 0.05) abundance of Bacteroidetes than NA, while NA had a significantly higher proportion (p < 0.05) of Firmicutes. Spirochaetest showed higher abundance in PD and PD_DM than in NA but was significantly higher only in PD (**Supplementary Figure 2**). Similar compositional patterns were observed at the bacterial genus level (**Figure 1D**).

### Oral microbiome difference and dysbiosis between groups

**Figure 2:**
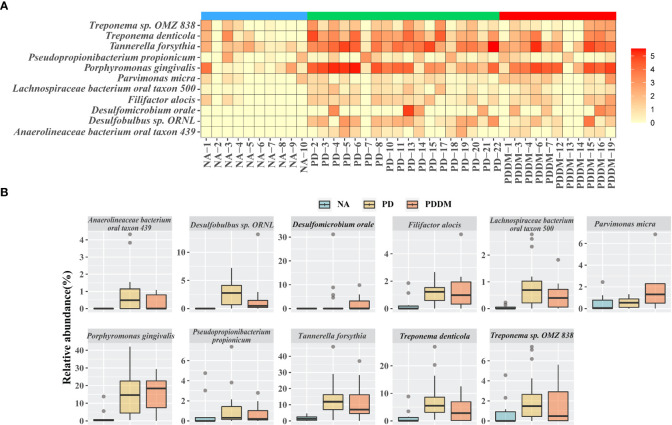
Key microbiome changes between NA and PD or PD_DM by differential abundance analysis. **(A)** Heatmap of the relative abundances (log2 transformed) and most abundantly present species in the PD or PD_DM. **(B)** Abundances of 11 significantly different bacterial species. Boxplots represent relative abundance of individual species in each group. A p-value < 0.05 is considered significant and the cutoff average relative abundance is > 0.5%.

To better understand the changes in the microbial community across groups, we performed differential abundance comparisons with ALDEx2 at the species level. We identified differentially abundant 39 and 28 taxa in the PD and PD_DM groups compared with the NA group. In addition, there were no significant differences between PD and PD_DM groups. (**Supplementary Tables 2** and **3**). Only species with an average relative abundance of 0.5 or higher were selected, and the distribution of relative abundance by disease state was confirmed. (**Figure 2A**). All selected strains showed a higher relative abundance in PD or PD_DM and were absent or rare in NA. *Porphyromonas gingivalis* and *Tannerella forsythia*, which are representative pathogenic bacteria of periodontitis, constituted more than 10% of PD and PD_DM (**Figure 2B**). The taxa *Haemophilus* sp. *Oral taxon 036*, *Streptococcus pseudopneumoniae*, *Streptococcus* sp. *A12*, and *Streptococcus* sp. *Oral taxon 431* existed at a low frequency, but they were almost absent in PD and PD_DM and could be identified only in NA (**Supplementary Figure 3**).

### Community structural changes according to disease status

**Figure 3:**
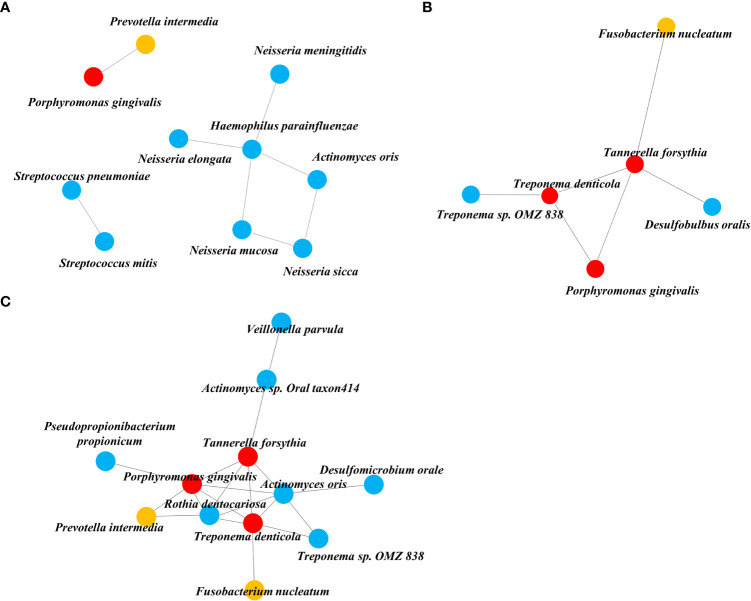
Co-occurrence network inferred from the SparCC algorithm applied to relative abundance species data. **(A)** NA, **(B)** PD, and **(C)** PD_DM. The orange circle represents the orange complex and the red circle represents the red complex.

We obtained three taxa networks in each clinical state i.e., “NA,” “PD,” and “PD_DM.” The co-occurrence patterns changed according to disease status; NA was the simplest, and PD_DM was the most complex (**Figure 3**). In NA, *Porphyromonas gingivalis* was included in the red complex co-occurring with *Prevotella intermedia*, which was included in the orange complex (**Figure 3A**). In PD, *Porphyromonas gingivalis* was connected to *Treponema deticola* and *Tannerella forsythia*, which were included in the red complex. Moreover, *Tannerella forsythia* co-occurred with *Fusobacterium nucleatum*, which was included in the orange complex (**Figure 3B**). The co-occurrence pattern of PD_DM was more complex. *Porphyromonas gingivalis* was linked to more strains than NA or PD. *Tannerella forsythia* and *Treponema denticola, which belonged* to the red complex and *Prevotella intermedia* and *Fusobacterium nucleatum*, which belonged to the orange complex, were also linked with more taxa (**Figure 3C**).

## Discussion

Periodontitis and DM have bidirectional relationships, and the simultaneous presence of these diseases is known to contribute to the pathophysiological mechanism of each disease, which worsens the disease condition (Sanz et al., 2018). Several studies have revealed that DM acts as an additional stress factor for microbial dysbiotic changes that occur during periodontitis progression (Long et al., 2017; Matsha et al., 2020; Sun et al., 2020). However, most of these studies were sampled in the surrounding environment, and not inside the tissue where periodontitis progresses, such as saliva, plaque, and gingival fluid. Our fundamental investigation, in line with previous studies, was whether alterations in the microbial community in the subgingival plaque of patients with periodontitis were caused by DM, and if it was the case within the tissue as well. Therefore, in this study, we used WGS data from gingival tissues. The oral microbiota was profiled by aligning reads that were not aligned with human references in the WGS data. Our results showed that certain environmental pressures can affect both the community structure and the members of the infiltrated ecosystem. This study first compared and analyzed the dysbiotic changes in the microbiotic community penetrating periodontal tissue according to the state of periodontitis and DM.

Alpha diversity analysis evaluates the richness and evenness of the oral microbiome (within-sample diversity), which not only considers how many species are present but also how evenly each species is distributed. Although it is intuitive to infer that a healthy group should increase alpha diversity, since a more diverse community is associated with greater resilience and healthier status from an ecological point of view (Proulx et al., 2010); however, our study did not find an increasing trend of alpha diversity in NA. Further, NA revealed a lower richness index than the PD and PD_DM groups. Periodontitis is caused by a complex alteration of the microbiome rather than by a few predominant pathogens. Thus, the microbiome does not become simpler with the onset of disease, but can become simpler as the number and load of pathogenic species increase, while the number and load of commensal species decrease (Zhang et al., 2021). Given that pathogens and commensals change simultaneously, it is difficult to determine the magnitude and direction of the changes in richness and evenness. It is more meaningful to assess beta diversity than alpha diversity in this study, as it can demonstrate how the microbiome community within tissues changes with periodontal and diabetic conditions. Our results showed that the NA group was different from PD and PD_DM, but there was no significant change in PD and PD_DM. Analysis of beta diversity and differentially abundant microbial profiles did not discriminate between PD and PD_DM. It is known that the morbidity of DM and periodontitis does not significantly affect the microbial diversity of subgingival plaques, and the macroscopic composition of bacteria infiltrating the tissues of patients with periodontitis is not significantly affected by DM. It is known that the comorbidity of periodontitis and DM does not significantly affect the microbial diversity of subgingival plaques (Farina et al., 2019), and it was confirmed that the regime shift of bacterial composition infiltrating the tissues of patients with periodontitis was not significantly affected by DM.

Through our network analysis results, it was confirmed that the red and orange complex, which are known to exist in the oral cavity of a healthy subject, also exist in the tissue of periodontally healthy subjects, but they do not form a connection with other bacteria and exist independently. *Porphyromonas gingivalis* is a representative periodontal pathogen, but its presence does not necessarily precipitate the disease condition, because its virulence factors (gingipains and lipid A phosphatases) can be controlled by intrinsic immune status (Hajishengallis, 2015). Therefore, we could reaffirm that periodontal pathogens need to form communities by connecting with accessory pathogens and pathobionts rather than their own existence to act as keystone pathogens in dysbiosis.

In the analysis of the microbial network in the tissue, six species were connected to each other in PD, but 12 species were connected in PD_DM, confirming that the PD_DM network was more complex and dense than PD. The microbiota belonging to the red and orange complexes, which induce excessive inflammation in periodontal tissue (Suzuki et al., 2013; Mohanty et al., 2019), showed a higher correlation with other species in PD_DM than in PD. Putative periodontal pathogens are enriched as the resident oral microbiota becomes dysbiotic, and inflammatory responses evoke tissue destruction, thus inducing an unremitting positive feedback loop of proteolysis, inflammation, and enrichment of periodontal pathogens (Sedghi et al., 2021). The high correlation between the pathogenic microbiota in PD_DM suggests that the inflammatory response due to this microbial imbalance might be more active in diabetic patients, which may accelerate periodontitis. This indicates that DM can contribute to a positive feedback loop, leading to periodontitis.

It is noteworthy that the network found in PD_DM is not completely different from that of PD and is an extension of the PD network including all six species. The local environment altered by DM did not cause an essential change in the composition of the microbial network in the tissue; rather, it expanded the existing network. In particular, it was found that *Actinomyces oralis* and *Rothia dentocariosa* participated in the core of this connecting structure, along with the three red complexes. Both are gram-positive, round to rod-shaped bacterial species that are part of the normal community of microbes residing in the mouth but are also known to be involved in the progression of periodontitis (Biyikoğlu et al., 2012; Kataoka et al., 2014). Both the microbes were not connected with the red and orange complexes for a healthy status or periodontitis alone; however, for DM, they further strengthened the network frame by connecting with other species at the core of the network. Some limitations of this study should be considered while interpreting the results. The small number of experimental samples and lack of experimental validation are considered limitations of our study. The above limitations can be overcome by increasing the sample size in future studies.

In conclusion, the current study suggests a potential mechanism of exacerbation of periodontitis caused by diabetes in context of the intra-gingival pathogenic bacterial network. During the progression of periodontitis, DM does not cause a regime shift for the microbial community formed by infiltration into the tissue; however, it can be expected that it will amplify the inflammatory response by further strengthening the bacterial network.

## Data availability statement

The original contributions presented in the study are publicly available. This data can be found on SRA: Accession number: SUB12266562.

## Ethics statement

The studies involving human participants were reviewed and approved by Institutional Review Board of Pusan National University Dental Hospital (PNUDH-2017-023). The patients/participants provided their written informed consent to participate in this study.

## Author contributions

YK and HP conceived and supervised the project. YY carried out data analysis and manuscript writing. H-JK wrote the original manuscript. JK, HL, and J-MS conducted the data analysis. All authors contributed to the article and approved the submitted version.

## Funding

This work was supported by the National Research Foundation of Korea (2018R1A5A2023879 and 2021R1A6A3A01086785).

## Conflict of interest

The authors declare that the research was conducted in the absence of any commercial or financial relationships that could be construed as a potential conflict of interest.

## Publisher’s note

All claims expressed in this article are solely those of the authors and do not necessarily represent those of their affiliated organizations, or those of the publisher, the editors and the reviewers. Any product that may be evaluated in this article, or claim that may be made by its manufacturer, is not guaranteed or endorsed by the publisher.

## Supplementary material

Supplementary Figure 1 NMDS visualization of beta diversity analysis based on the weighted and unweighted UniFrac distance matrices.

Supplementary Figure 2 Boxplots describe the relative abundance of phyla between NA and PD or PD_DM.

Supplementary Figure 3 Significant differences in taxonomic abundance that discriminate NA and PD or PD_DM at the species levels.
