# Supplementary figures and images for: Differential microbiota network in gingival tissues between periodontitis and periodontitis with diabetes
Source: Front Cell Infect Microbiol. 2022 Dec 2;12:1061125. doi: 10.3389/fcimb.2022.1061125 (PMC9755495; doi:10.3389/fcimb.2022.1061125)

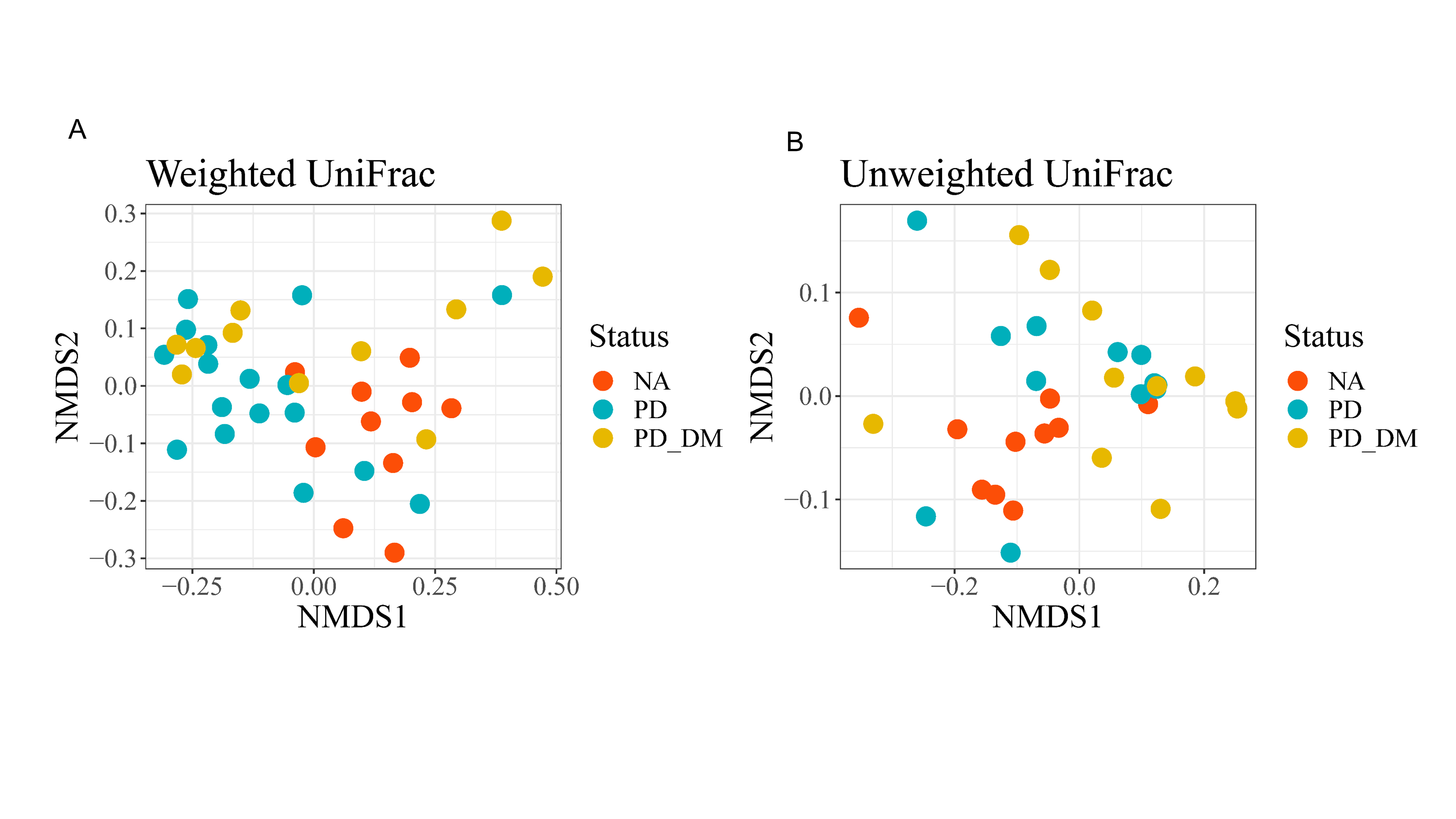

Supplement: Supplementary Figure 1 — NMDS visualization of beta diversity analysis based on the weighted and unweighted UniFrac distance matrices. [file Image_1.tif]

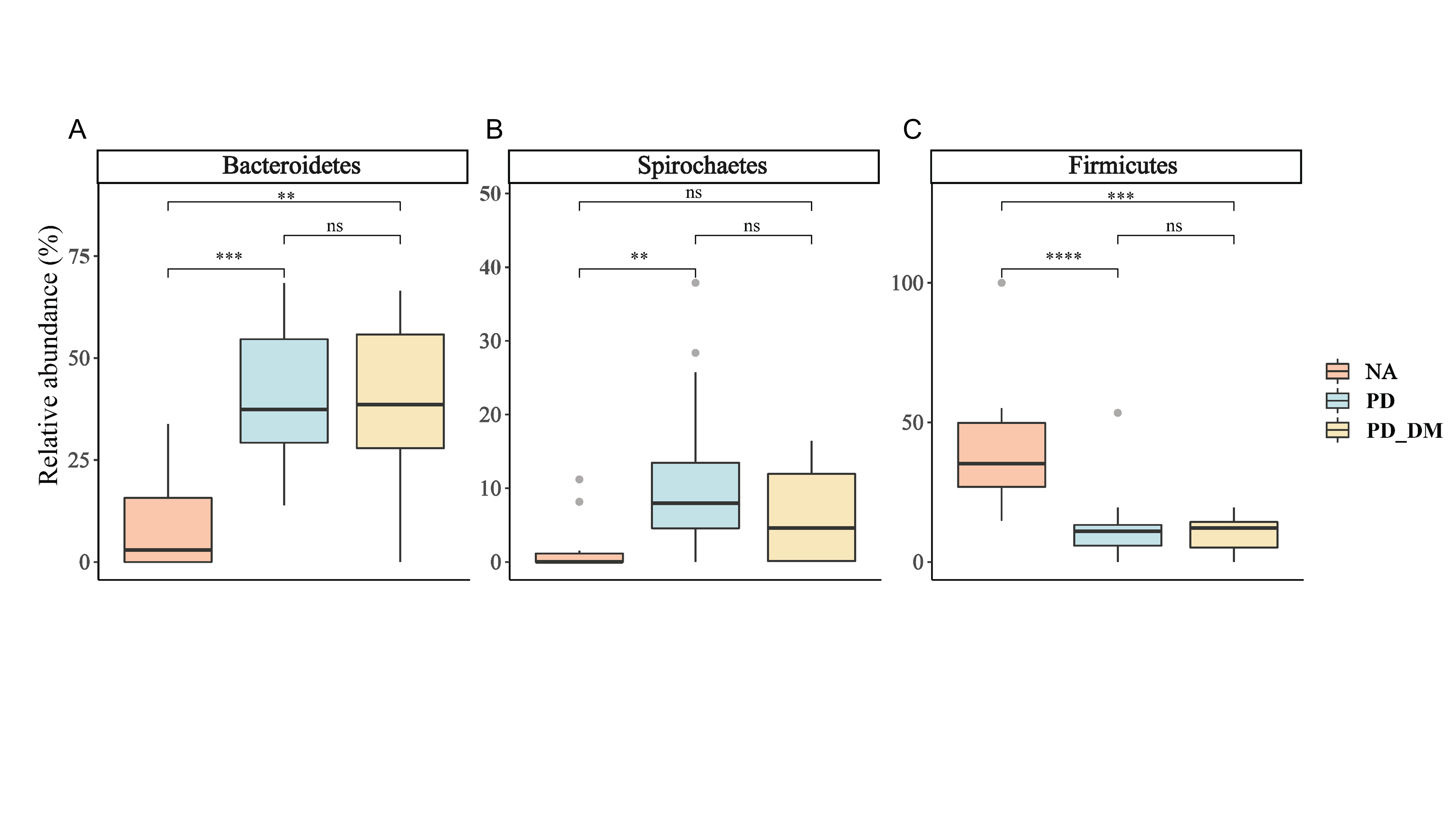

Supplement: Supplementary Figure 2 — Boxplots describe the relative abundance of phyla between NA and PD or PD_DM. [file Image_2.tif]

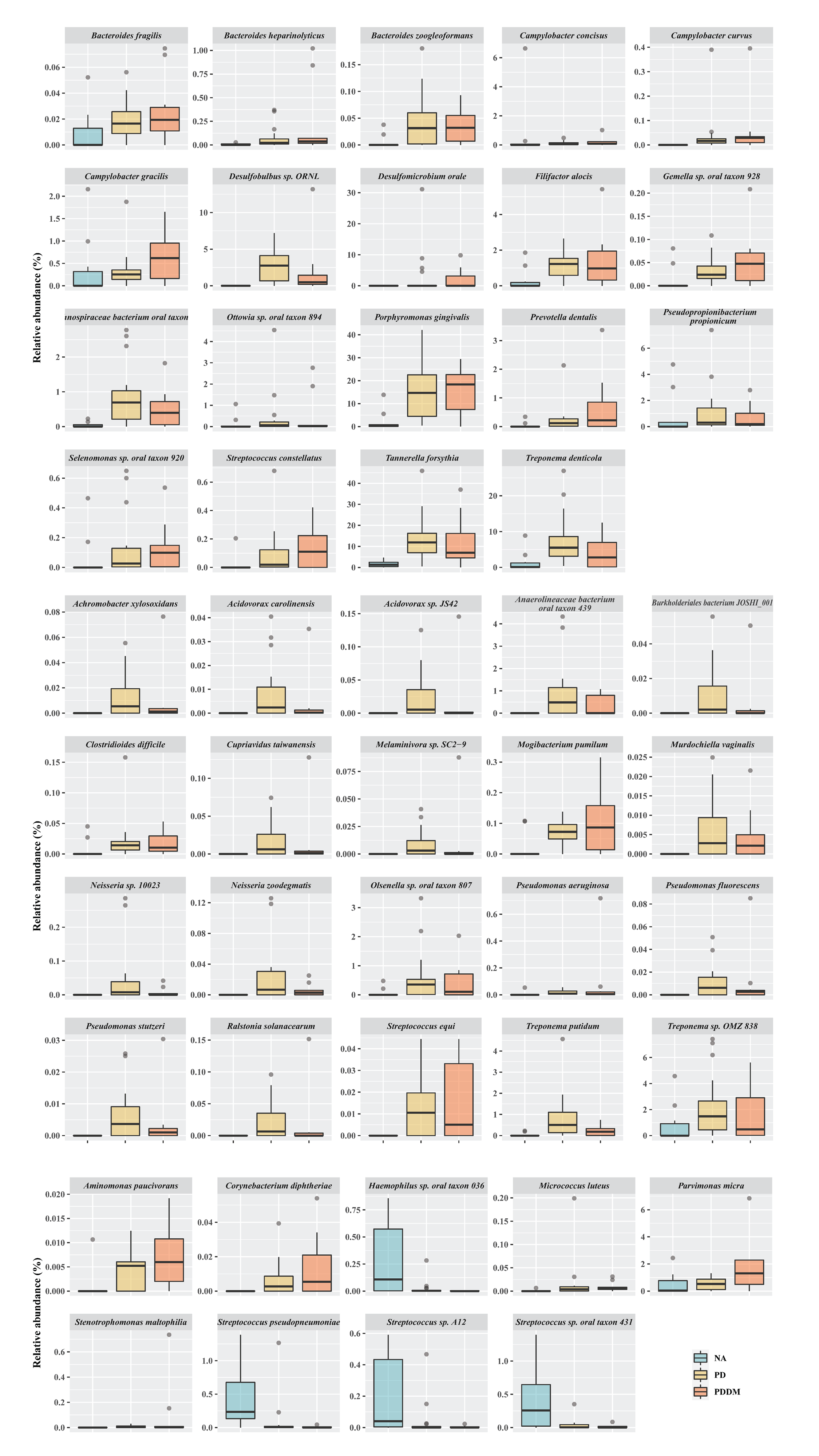

Supplement: Supplementary Figure 3 — Significant differences in taxonomic abundance that discriminate NA and PD or PD_DM at the species levels. [file Image_3.tif]
