# Supplementary material for: Differential microbiota network in gingival tissues between periodontitis and periodontitis with diabetes
Source: Front Cell Infect Microbiol. 2022 Dec 2;12:1061125. doi: 10.3389/fcimb.2022.1061125 (PMC9755495; doi:10.3389/fcimb.2022.1061125)
Supplement: Supplementary file 4 [file Table_1.docx]

| **Supplementary Table 1.** Summary of the read counts for all patients. | | | | |
| --- | --- | --- | --- | --- |
| **Sample identifier** | **Total read** | **Microbiome read** | **Assigned taxa** | **Reads assigned to microbiome (%)** |
| NA-1 | 351893726 | 546644 | 147 | 0.16 |
| NA-2 | 357600002 | 459945 | 116 | 0.13 |
| NA-3 | 339021017 | 1003826 | 320 | 0.3 |
| NA-4 | 385158738 | 498172 | 139 | 0.13 |
| NA-5 | 351514970 | 257617 | 90 | 0.07 |
| NA-6 | 385776472 | 393295 | 91 | 0.1 |
| NA-7 | 382237445 | 627188 | 167 | 0.16 |
| NA-8 | 365669759 | 388292 | 127 | 0.11 |
| NA-9 | 344895638 | 296213 | 66 | 0.09 |
| NA-10 | 403665542 | 377750 | 117 | 0.09 |
| NP-2 | 372439071 | 706552 | 146 | 0.19 |
| NP-3 | 362821914 | 8905361 | 1712 | 2.45 |
| NP-4 | 374547731 | 3579034 | 698 | 0.96 |
| NP-5 | 350130366 | 7164514 | 1162 | 2.05 |
| NP-6 | 327957986 | 2386716 | 318 | 0.73 |
| NP-7 | 349879921 | 471585 | 91 | 0.13 |
| NP-8 | 345490966 | 1878968 | 349 | 0.54 |
| NP-10 | 359986282 | 31538576 | 2594 | 8.76 |
| NP-11 | 365926000 | 6091065 | 1346 | 1.66 |
| NP-13 | 387870481 | 711245 | 81 | 0.18 |
| NP-14 | 388017366 | 2617187 | 947 | 0.67 |
| NP-15 | 355081918 | 273897 | 76 | 0.08 |
| NP-17 | 332857054 | 477654 | 128 | 0.14 |
| NP-18 | 386680430 | 22242360 | 2543 | 5.75 |
| NP-19 | 338481577 | 12576264 | 2028 | 3.72 |
| NP-20 | 368124360 | 1844077 | 546 | 0.5 |
| NP-21 | 359060063 | 1840526 | 839 | 0.51 |
| NP-22 | 371058337 | 800002 | 145 | 0.22 |
| NPDM-1 | 375930623 | 656546 | 189 | 0.17 |
| NPDM-3 | 396268950 | 57730539 | 3004 | 14.57 |
| NPDM-4 | 375551479 | 33417855 | 2574 | 8.9 |
| NPDM-6 | 351090364 | 645073 | 113 | 0.18 |
| NPDM-7 | 377447659 | 22804822 | 2354 | 6.04 |
| NPDM-12 | 366724145 | 27192657 | 2249 | 7.42 |
| NPDM-13 | 377681840 | 1151812 | 675 | 0.3 |
| NPDM-14 | 358449103 | 11199 | 69 | 0.01 |
| NPDM-15 | 366296139 | 2025038 | 309 | 0.55 |
| NPDM-16 | 375163324 | 4137056 | 512 | 1.1 |
| NPDM-19 | 359775564 | 631256 | 131 | 0.18 |

| **Supplementary Table 2.** Significantly different taxa between NA and PD. | | | | | |
| --- | --- | --- | --- | --- | --- |
| **Species** | **diff.btw** | **effect** | **overlap** | **p-value** | **BH** |
| *Desulfobulbus sp. ORNL* | 14.109 | 2.698 | 0.055 | 0 | 0.024 |
| *Anaerolineaceae bacterium oral taxon 439* | 11.318 | 0.974 | 0.19 | 0.018 | 0.162 |
| *Treponema putidum* | 10.688 | 1.269 | 0.105 | 0 | 0.054 |
| *Filifactor alocis* | 9.325 | 1.038 | 0.153 | 0.002 | 0.092 |
| *Olsenella sp. oral taxon 807* | 9.144 | 0.932 | 0.194 | 0.011 | 0.149 |
| *Campylobacter gracilis* | 9.098 | 0.886 | 0.215 | 0.014 | 0.173 |
| *Treponema sp. OMZ 838* | 8.904 | 0.791 | 0.187 | 0.007 | 0.137 |
| *Treponema denticola* | 8.454 | 1.103 | 0.11 | 0 | 0.06 |
| *Lachnospiraceae bacterium oral taxon 500* | 8.258 | 1.035 | 0.116 | 0.001 | 0.062 |
| *Prevotella dentalis* | 7.995 | 0.872 | 0.213 | 0.017 | 0.177 |
| *Mogibacterium pumilum* | 7.937 | 1.078 | 0.175 | 0.005 | 0.119 |
| *Pseudopropionibacterium propionicum* | 7.697 | 0.722 | 0.254 | 0.035 | 0.239 |
| *Campylobacter concisus* | 7.514 | 0.777 | 0.256 | 0.044 | 0.262 |
| *Streptococcus constellatus* | 7.263 | 0.929 | 0.18 | 0.008 | 0.129 |
| *Campylobacter curvus* | 7.172 | 1.274 | 0.114 | 0.001 | 0.062 |
| *Gemella sp. oral taxon 928* | 6.799 | 1.024 | 0.18 | 0.005 | 0.124 |
| *Selenomonas sp. oral taxon 920* | 6.715 | 0.847 | 0.223 | 0.018 | 0.178 |
| *Ottowia sp. oral taxon 894* | 6.548 | 0.869 | 0.182 | 0.007 | 0.134 |
| *Porphyromonas gingivalis* | 6.472 | 1.175 | 0.089 | 0 | 0.043 |
| *Bacteroides zoogleoformans* | 6.344 | 0.773 | 0.207 | 0.016 | 0.172 |
| *Streptococcus equi* | 6.235 | 1.025 | 0.166 | 0.008 | 0.124 |
| *Cupriavidus taiwanensis* | 6.06 | 0.93 | 0.192 | 0.016 | 0.157 |
| *Acidovorax sp. JS42* | 6.059 | 0.879 | 0.207 | 0.021 | 0.174 |
| *Pseudomonas aeruginosa* | 6.025 | 0.975 | 0.193 | 0.011 | 0.146 |
| *Bacteroides heparinolyticus* | 5.911 | 0.965 | 0.131 | 0.001 | 0.08 |
| *Neisseria sp. 10023* | 5.769 | 0.751 | 0.244 | 0.041 | 0.223 |
| *Neisseria zoodegmatis* | 5.731 | 0.883 | 0.206 | 0.023 | 0.178 |
| *Ralstonia solanacearum* | 5.715 | 0.821 | 0.222 | 0.04 | 0.211 |
| *Achromobacter xylosoxidans* | 5.579 | 0.887 | 0.196 | 0.017 | 0.157 |
| *Clostridioides difficile* | 5.475 | 0.774 | 0.229 | 0.023 | 0.196 |
| *Tannerella forsythia* | 5.383 | 1.337 | 0.079 | 0 | 0.039 |
| *Desulfomicrobium orale* | 5.349 | 0.694 | 0.198 | 0.02 | 0.167 |
| *Melaminivora sp. SC2-9* | 5.331 | 0.963 | 0.161 | 0.006 | 0.112 |
| *Bacteroides fragilis* | 5.242 | 0.806 | 0.188 | 0.008 | 0.142 |
| *Acidovorax carolinensis* | 5.135 | 0.943 | 0.168 | 0.007 | 0.116 |
| *Murdochiella vaginalis* | 5.131 | 0.893 | 0.174 | 0.009 | 0.125 |
| *Pseudomonas fluorescens* | 5.096 | 0.85 | 0.199 | 0.02 | 0.165 |
| *Pseudomonas stutzeri* | 5.028 | 0.947 | 0.17 | 0.007 | 0.116 |
| *Burkholderiales bacterium JOSHI_001* | 5.001 | 0.862 | 0.186 | 0.016 | 0.156 |

| **Supplementary Table 3.** Significantly different taxa between NA and PD_DM. | | | | | |
| --- | --- | --- | --- | --- | --- |
| **Species** | **diff.btw** | **effect** | **overlap** | **p-value** | **BH** |
| *Desulfobulbus sp. ORNL* | 12.126 | 1.325 | 0.148 | 0.01 | 0.254 |
| *Campylobacter gracilis* | 9.704 | 0.845 | 0.225 | 0.037 | 0.36 |
| *Desulfomicrobium orale* | 9.122 | 1.251 | 0.087 | 0.002 | 0.175 |
| *Prevotella dentalis* | 9.04 | 0.856 | 0.218 | 0.037 | 0.349 |
| *Pseudopropionibacterium propionicum* | 8.382 | 0.755 | 0.223 | 0.035 | 0.361 |
| *Streptococcus constellatus* | 8.304 | 0.893 | 0.208 | 0.041 | 0.348 |
| *Filifactor alocis* | 8.18 | 0.758 | 0.208 | 0.028 | 0.339 |
| *Campylobacter concisus* | 7.995 | 0.833 | 0.233 | 0.041 | 0.372 |
| *Campylobacter curvus* | 7.583 | 1.207 | 0.142 | 0.009 | 0.247 |
| *Selenomonas sp. oral taxon 920* | 7.487 | 0.804 | 0.224 | 0.045 | 0.369 |
| *Gemella sp. oral taxon 928* | 7.086 | 0.875 | 0.2 | 0.024 | 0.324 |
| *Porphyromonas gingivalis* | 7.05 | 1.093 | 0.138 | 0.003 | 0.234 |
| *Lachnospiraceae bacterium oral taxon 500* | 6.509 | 0.758 | 0.198 | 0.02 | 0.312 |
| *Bacteroides heparinolyticus* | 6.491 | 0.879 | 0.149 | 0.007 | 0.251 |
| *Bacteroides zoogleoformans* | 6.198 | 0.787 | 0.207 | 0.033 | 0.34 |
| *Ottowia sp. oral taxon 894* | 5.996 | 0.797 | 0.23 | 0.043 | 0.377 |
| *Treponema denticola* | 5.873 | 0.591 | 0.234 | 0.042 | 0.374 |
| *Corynebacterium diphtheriae* | 5.675 | 0.937 | 0.178 | 0.028 | 0.305 |
| *Micrococcus luteus* | 5.325 | 1.049 | 0.134 | 0.005 | 0.231 |
| *Bacteroides fragilis* | 5.226 | 0.724 | 0.209 | 0.028 | 0.335 |
| *Stenotrophomonas maltophilia* | 5.195 | 0.926 | 0.159 | 0.016 | 0.271 |
| *Aminomonas paucivorans* | 5.052 | 0.852 | 0.185 | 0.022 | 0.302 |
| *Parvimonas micra* | 5.031 | 0.797 | 0.134 | 0.004 | 0.236 |
| *Tannerella forsythia* | 5.013 | 0.976 | 0.162 | 0.008 | 0.267 |
| *Streptococcus sp. oral taxon 431* | -5.093 | -0.618 | 0.228 | 0.046 | 0.375 |
| *Streptococcus sp. A12* | -5.262 | -0.7 | 0.196 | 0.024 | 0.317 |
| *Haemophilus sp. oral taxon 036* | -5.308 | -0.726 | 0.224 | 0.041 | 0.363 |
| *Streptococcus pseudopneumoniae* | -5.5 | -0.972 | 0.104 | 0.001 | 0.2 |
